# TTK, CDC25A, and ESPL1 as Prognostic Biomarkers for Endometrial Cancer

**DOI:** 10.1155/2020/4625123

**Published:** 2020-11-17

**Authors:** Qiannan Yang, Bojun Yu, Jing Sun

**Affiliations:** Department of Gynaecology, Shanghai First Maternity and Infant Hospital, Tongji University School of Medicine, Shanghai /201204, China

## Abstract

**Objective:**

Endometrial cancer (EC) is one of the most common malignant gynaecological tumours worldwide. This study was aimed at identifying EC prognostic genes and investigating the molecular mechanisms of these genes in EC.

**Methods:**

Two mRNA datasets of EC were downloaded from the Gene Expression Omnibus (GEO). The GEO2R tool and Draw Venn Diagram were used to identify differentially expressed genes (DEGs) between normal endometrial tissues and EC tissues. Then, Gene Ontology (GO) and Kyoto Encyclopedia of Genes and Genomes (KEGG) pathway analyses were performed using the Database for Annotation, Visualization and Integrated Discovery (DAVID). Next, the protein-protein interactions (PPIs) of these DEGs were determined by the Search Tool for the Retrieval of Interacting Genes (STRING) tool and Cytoscape with Molecular Complex Detection (MCODE). Furthermore, Kaplan-Meier survival analysis was performed by UALCAN to verify genes associated with significantly poor prognosis. Next, Gene Expression Profiling Interactive Analysis (GEPIA) was used to verify the expression levels of these selected genes. Additionally, a reanalysis of the KEGG pathways was performed to understand the potential biological functions of selected genes. Finally, the associations between these genes and clinical features were analysed based on TCGA cancer genomic datasets for EC.

**Results:**

In EC tissues, compared with normal endometrial tissues, 147 of 249 DEGs were upregulated and 102 were downregulated. A total of 64 upregulated genes were assembled into a PPI network. Next, 14 genes were found to be both associated with significantly poor prognosis and highly expressed in EC tissues. Reanalysis of the KEGG pathways found that three of these genes were enriched in the cell cycle pathway. *TTK*, *CDC25A*, and *ESPL1* showed higher expression in cancers with late stage and higher tumour grade.

**Conclusion:**

In summary, through integrated bioinformatics approaches, we found three significant prognostic genes of EC, which might be potential therapeutic targets for EC patients.

## 1. Introduction

Endometrial cancer (EC) is the most common gynaecological malignancy, with an increasing incidence in North America, Europe, and more than 20 developed countries elsewhere [1, 2]. There were 61 880 new cases and 12 160 deaths from EC in the United States in 2019 [3], up from 60 050 new cases and 10 470 deaths in 2016 [4]. Two histological classifications have been described among endometrioid adenocarcinoma, type I and type II, and seventy to eighty percent of new cases are type I. Type I tumours, which are mostly of lower grade, are mediated by oestrogen and have endometrioid histology. Type II tumours are composed of higher-grade tumours (generally clear cell or serous) and are more common in thinner and older women [5, 6]. The prognosis of EC patients is related to the stage and grade of the disease. EC patients with stage III or IV endometrial cancer typically have a worse prognosis, and patients with stages I and II have a better survival [7]. Thus, finding more credible prognostic biomarkers is important for improving the therapies for EC, and investigating their role development is important for further elucidation of the underlying mechanisms of EC.

The Gene Expression Omnibus (GEO) database gathers next-generation sequencing data and high-throughput microarrays submitted by the research community constructed and kept by the National Center for Biotechnology Information (NCBI). Screening for differentially expressed genes (DEGs), analysis of gene regulatory networks, and exploration of molecular signals and interrelationships can be performed via the GEO database. Thousands of DEGs that may be involved in EC development have been screened from a large number of studies of gene expression profiles of EC in GEO. However, the identification of DEGs in multiple studies has been limited as a result of the different technology platforms used, the different treatment of data across studies, and the heterogeneity of tissues or samples in different independent experiments. We comprehensively analysed microarray data from two gene expression profiling experiments with consideration of the false positive probability of microarray results.

Two initial gene expression profiles (GSE17025 and GSE63678) were downloaded from the GEO database. To identify common DEGs, the GEO2R tool was applied to integrate the expression profiles. Next, Gene Ontology (GO) and Kyoto Encyclopedia of Genes and Genomes (KEGG) pathway analyses were performed using the Database for Annotation, Visualization and Integrated Discovery (DAVID). The Search Tool for the Retrieval of Interacting Genes (STRING) database was then applied to create a protein-protein interaction (PPI) network. Later, the core genes among the DEGs were identified by Cytotype Molecular Complex Detection (MCODE). Additionally, these core DEGs were explored by UALCAN to obtain information on patient prognosis (*P* < 0.05). Moreover, the expression of DEGs between normal endometrial samples and EC samples was validated by Gene Expression Profiling Interactive Analysis (GEPIA) (*P* < 0.01). Then, KEGG pathway enrichment of these designated DEGs was reanalysed by DAVID. *TTK*, *CDC25A*, and *ESPL1* were found to be significantly enriched in the cell cycle pathway. Last, *TTK*, *CDC25A*, and *ESPL1* showed higher expression in the late cancer stage and higher tumour grade. In conclusion, *TTK*, *CDC25A*, and *ESPL1* are associated with poor prognosis in EC and could be potential therapeutic biomarkers that might be beneficial for EC patients.

## 2. Materials and Methods

### 2.1. Microarray Data Information

GEO (http://www.ncbi.nlm.nih.gov/geo) is an open database of microarray/gene profiles [8]. Two mRNA expression datasets containing microarray data from normal endometrial tissues and EC tissue, GSE63678 [9] and GSE17025 [10], were selected. The GSE63678 microarray data included 5 normal endometrial tissues and 7 EC tissues and were generated using the GPL571 platform Affymetrix Human Genome U133A 2.0 Array. The GSE17025 microarray data included 12 normal endometrial tissues and 91 EC tissues and were generated using the GPL570 platform Affymetrix Human Genome U133 Plus 2.0 Array.

### 2.2. Identification of DEGs

GEO2R is an interactive web tool that allows users to compare two or more groups of samples in a GEO series to identify genes that are differentially expressed across experimental conditions. The GEO2R online tool (http://www.ncbi.nlm.nih.gov/geo/geo2r/) was applied to recognize DEGs between normal endometrial tissues and EC tissues with an adjusted *P* value < 0.05 and ∣log_2_FC | >1. The Draw Venn Diagram (http://bioinformatics.psb.ugent.be/webtools/Venn/) can be used to calculate the intersections of the list of elements. Therefore, we used this tool to analyse the raw data in TXT format and obtain the DEGs in the two datasets. Upregulated genes were DEGs with log_2_FC > 1, while downregulated genes were DEGs with log_2_FC < ‐1.

### 2.3. Analysis of GO and KEGG Pathway Enrichment

GO is a general annotation method for genes and their RNA or protein products that is used to characterize the biological characteristics of high-throughput genomic data [11]. KEGG is a compendium of databases with information on genomes, chemical materials, biological pathways, diseases, and drugs [12]. As an online bioinformatics tool, DAVID (http://david.ncifcrf.gov/) is aimed at recognizing the functions of genes or proteins [13]. The DEG enrichment of GO terms (biological process (BP), cell component (CC), and molecular function (MF)) and KEGG pathways was visualized by DAVID (*P* < 0.05) [14].

### 2.4. Analysis of PPI Network and Module

As an online tool, STRING (http://string-db.org/) is designed to visualize PPI information [15]. The potential relationships among DEGs were identified by the STRING app in Cytoscape (confidencescore ≥ 0.4 and maximum number of interactors = 0). Furthermore, modules of the PPI network were validated via the MCODE app in Cytoscape (max.depth = 100, nodescorecut − off = 0.2, degreecut − off = 2, and *k*‐score = 2) [16].

### 2.5. Survival Analysis and mRNA Expression of Core Genes

The expression level of various genes has an impact on patient survival, which can be shown by Kaplan-Meier plots. As a friendly, comprehensive, and interactive web resource for data analysis of cancer omics, UALCAN (http://ualcan.path.uab.edu/index.html) was applied to obtain Kaplan-Meier plots and gene expression profiles based on TCGA data [17]. The significance of survival impact is measured by the log rank test (*P* < 0.05). In addition, based on the TCGA and GTEx projects, the level of gene expression in normal endometrial samples and EC samples was validated via GEPIA (|log_2_FC | cut‐off = 1, *P* value cut‐off = 0.01) [18]. Finally, the University of California Santa Cruz (UCSC) Xena website (http://xena.ucsc.edu/), an analytics, visualization, and universal integration tool for analysing and viewing public datasets was used to assess TCGA cancer genomic datasets for EC. We analysed the associations between prognostic genes and clinical features (cancer stage and tumour grade) by *t* test (*P* < 0.05). The cBio Cancer Genomics Portal (cBioPortal) is a publicly accessible resource (http://www.cbioportal.org/) [19, 20]. We downloaded clinical datasets and cancer genomic datasets for EC [21] from the cBioPortal website and analysed the correlations between prognostic gene expression and survival in EC, and the significance of survival impact is measured by the log rank test (*P* < 0.05).

## 3. Results

### 3.1. Identification of DEGs in EC

**Figure 1:**
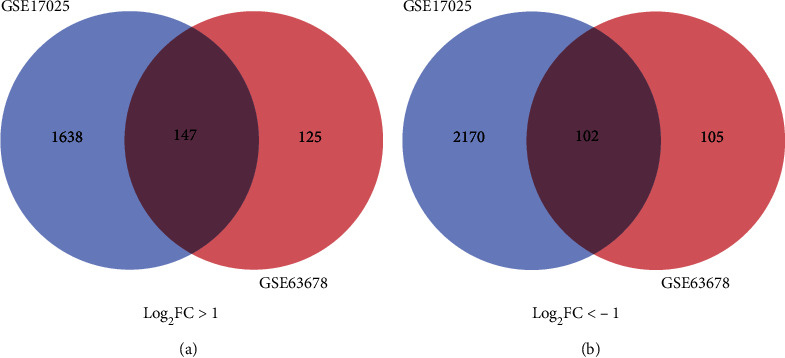
Validation of 249 DEGs in the two datasets (GSE63678 and GSE17025) via Draw Venn Diagram. (a) 147 upregulated DEGs in the two datasets (log_2_FC > 1). (b) 102 downregulated DEGs in two datasets (log_2_FC < −1).

Our study included 17 normal endometrial tissues and 98 EC tissues. A total of 4 057 and 479 DEGs were extracted from GSE17025 and GSE63678 using the GEO2R online tool. Then, the DEGs common to the two datasets were identified via the Draw Venn Diagram. Compared with normal endometrial tissues, 249 common DEGs were discovered, of which 147 were upregulated (adjusted *P* value < 0.05, log_2_FC > 1) and 102 were downregulated (adjusted *P* value < 0.05, log_2_FC < ‐1) in EC tissues (Figure 1 and Table 1).

### 3.2. GO and KEGG Pathway Analysis of DEGs in EC

**Figure 2:**
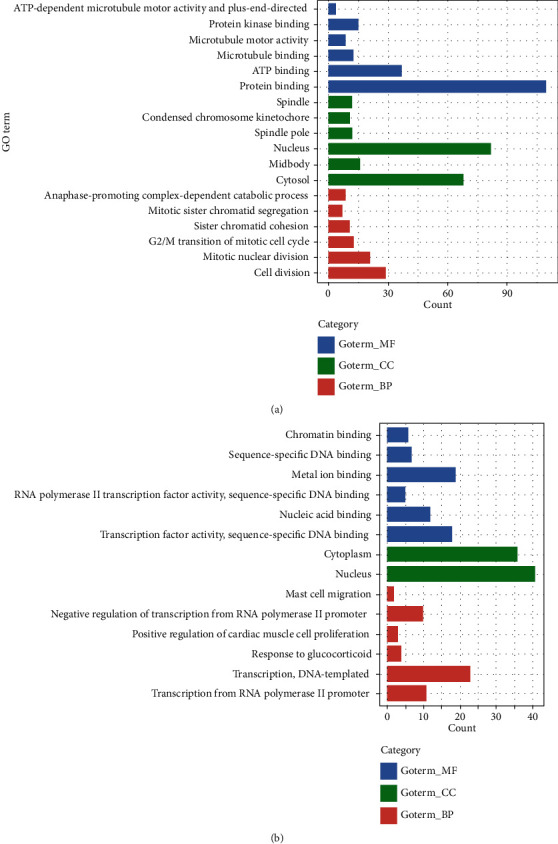
The GO term analysis of the 249 DEGs: (a) upregulated gene enrichment in GO; (b) downregulated gene enrichment in GO. GO: Gene Ontology; DEGs: differentially expressed genes.

**Figure 3:**
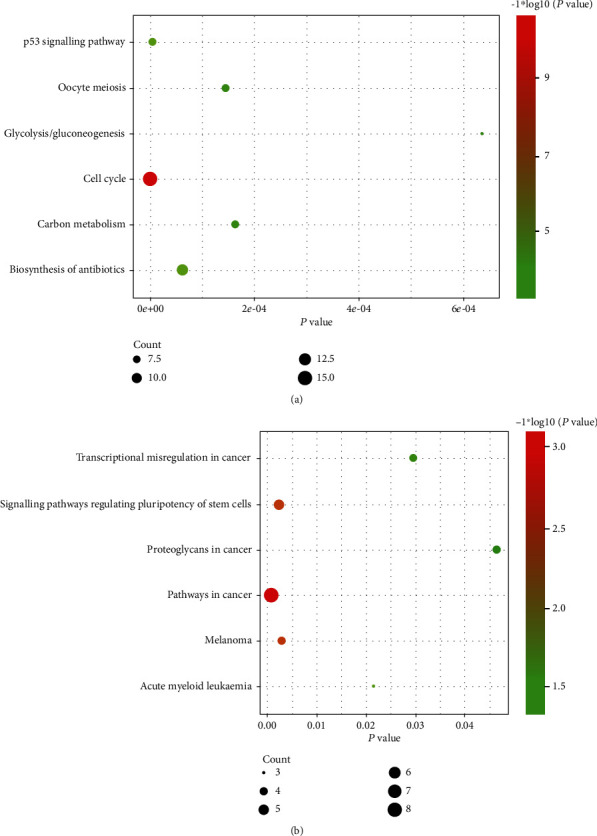
The KEGG pathway analysis of the 249 DEGs. (a) Upregulated genes enrichment in KEGG pathway. (b) Downregulated genes enrichment in KEGG pathway. KEGG, Kyoto Encyclopedia of Genes and Genomes; DEGs, differentially expressed genes.

The GO and KEGG pathway analysis of 249 DEGs was performed via DAVID. The top six GO terms in the BP, CC, and MF categories are shown in Figure 2. The upregulated DEGs were mainly enriched in cell division among the BP categories, the nucleus among the CC categories, and protein binding among the MF categories. The downregulated DEGs were mainly associated with transcription, DNA-templated among the BP categories, nucleus among the CC categories, and metal ion binding among the MF categories. The KEGG pathway analysis revealed that the upregulated DEGs were significantly enriched in the cell cycle, while the downregulated DEGs were mainly enriched in pathways in cancer (Figure 3).

### 3.3. Analysis of PPI and Module

**Figure 4:**
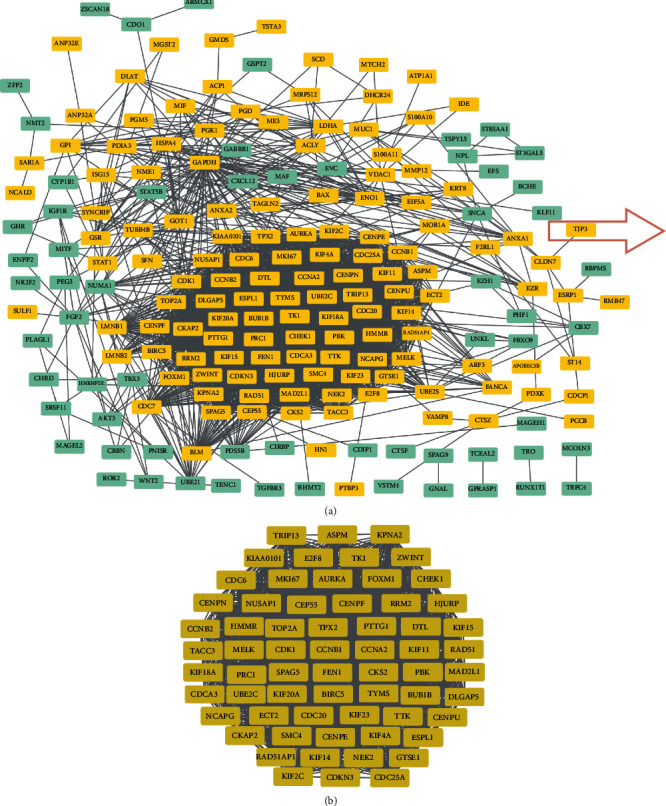
STRING and module analysis-built DEG PPI network. (a) The DEG PPI network complex had a total of 197 DEGs. The edges mean the interaction between proteins, the nodes mean proteins, and upregulated DEGs are represented by yellow rectangles and downregulated DEGs are represented by green rectangles. (b) Module analysis through the Cytoscape app (*k*‐core = 2, max.depth = 100, nodescorecut‐off = 0.2, and degreecut‐off = 2).

Among 249 DEGs, 197 DEGs were assembled into a DEG PPI network complex comprising 2 551 edges and 197 nodes, of which 65 were downregulated and 132 were upregulated (Figure 4(a)). The DEG PPI network excluded a total of 52 DEGs (Figure 4(a)). Next, further analysis using Cytotype MCODE revealed that 64 central nodes of the 197 nodes were all upregulated genes (Figure 4(b)).

### 3.4. Analysis of Core Genes via UALCAN and GEPIA

**Figure 5:**
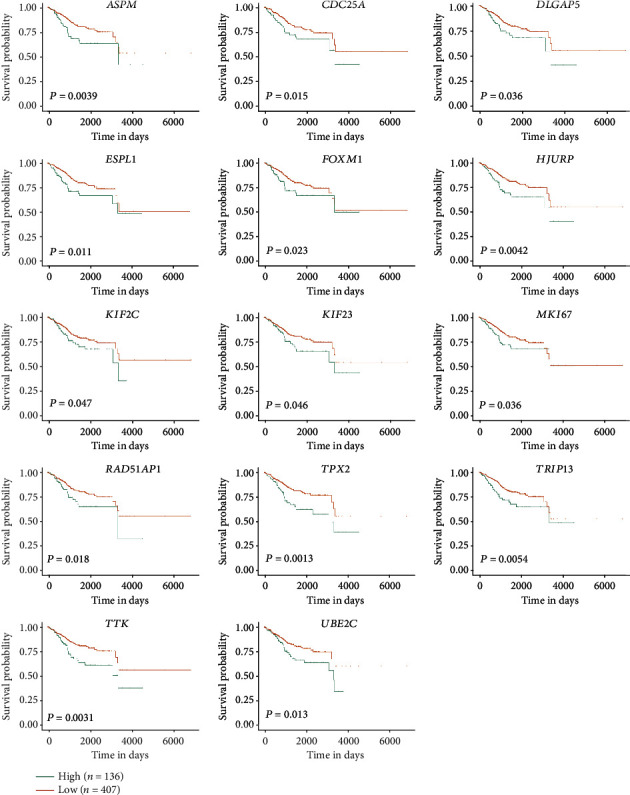
The Kaplan-Meier plots of the 64 core genes. Using the UALCAN online tool to verify the survival curves of the 64 core genes, and the survival rate of 14 of 64 genes was significantly poor (*P* < 0.05).

**Figure 6:**
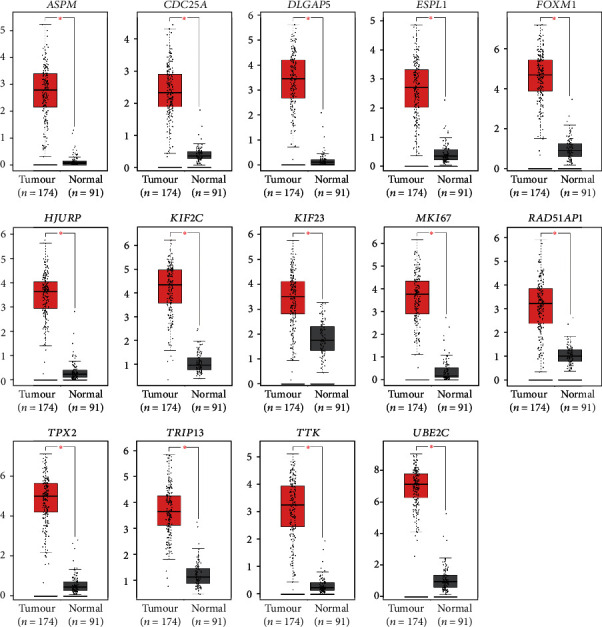
The expression of 14 genes was significantly high in endometrial cancer. The GEPIA website was applied to analyse 14 genes that were associated with poor prognosis. All of 14 genes highly expressed in endometrial cancer samples contrasted to normal samples (^∗^*P* < 0.01). Tumour tissues represented by red colour and normal tissues represented by grey colour.

UALCAN was applied to analyse the survival data for 64 core genes. The results showed that 14 genes (*TRIP13*, *MKI67*, *UBE2C*, *RAD51AP1*, *DLGAP5*, *TTK*, *KIF23*, *TPX2*, *ESPL1*, *FOXM1*, *HJURP*, *KIF2C*, *CDC25A*, and *ASPM*) were associated with significantly poorer survival, whereas 50 genes showed no significant correlations (*P* < 0.05, Figure 5). Next, we used GEPIA to examine the gene expression level of the 14 genes between normal endometrial specimens and EC specimens. The results indicated that all 14 genes were overexpressed in EC samples compared with normal endometrial samples (*P* < 0.01, Figure 6).

### 3.5. KEGG Reanalysis of 14 Designated Genes by DAVID

**Figure 7:**
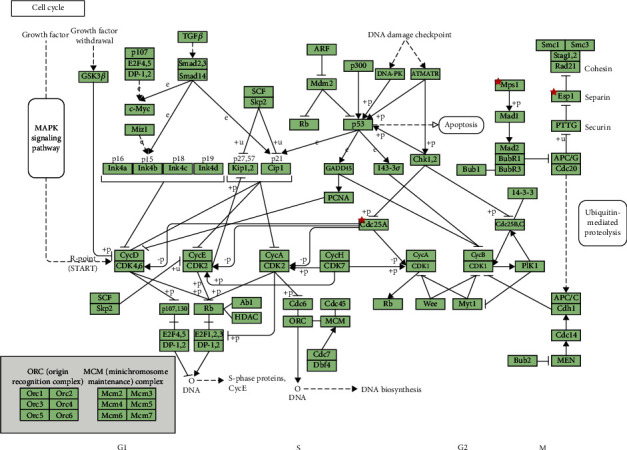
Reanalysis of 14 designated genes via KEGG pathway enrichment. Three genes (*TTK*, *CDC25A*, and *ESPL1*) were markedly concentrated in the cell cycle pathway. *Eps1* means *ESPL1*. *Cdc25A* means *CDC25A*. *Mps1* means *TTK*.

Reanalysis of the KEGG pathway data was performed using DAVID to understand the potential biological functions of these 14 designated DEGs (*P* < 0.05). We found that three genes (*TTK*, *CDC25A*, and *ESPL1*) were meaningfully enriched in the cell cycle pathway (*P* = 0.0019, Figure 7).

### 3.6. Correlation Analysis between *TTK*, *CDC25A*, and *ESPL1* Expression and Clinical Features

**Figure 8:**
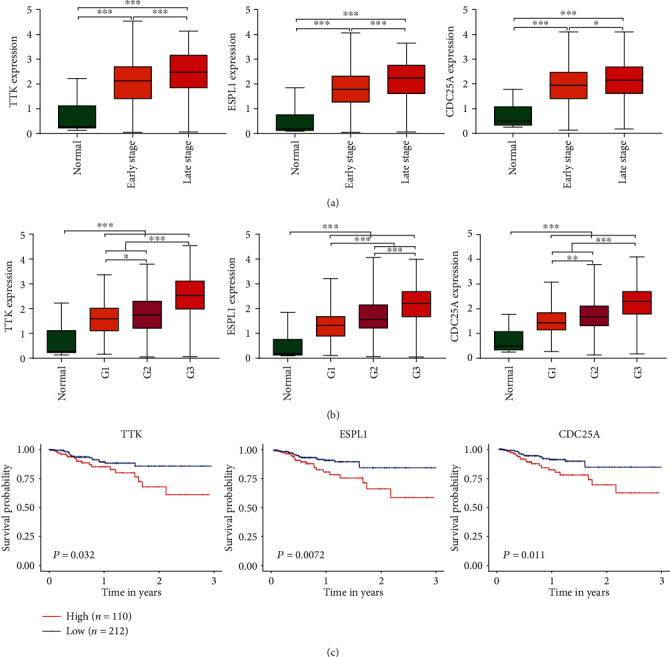
Analysis between *TTK*, *CDC25A*, and *ESPL1* expression and clinical features of EC. (a) Correlation analysis between *TTK*, *CDC25A*, and *ESPL1* expression with EC stage; early stage means stage I and stage II; late stage means stage III and stage IV. (b) Correlation analysis between *TTK*, *CDC25A*, and *ESPL1* expression with tumour grade. (c) Correlation between *TTK*, *CDC25A*, and *ESPL1* expression and survival of EC.

We analysed cancer genomic datasets from the UCSC Xena website and found that *TTK*, *CDC25A*, and *ESPL1* were highly expressed in EC tissues compared with normal tissues. In addition, *TTK*, *CDC25A*, and *ESPL1* showed higher expression in cancers with late stage and higher tumour grade, compared with early stage and lower tumour grade (*P* < 0.05, Figures 8(a) and 8(b)). Clinical datasets and cancer genomic datasets from the cBioPortal website were analysed, and the results showed that in cases with *TTK*, *CDC25A*, and *ESPL1* amplification, EC patients had worse survival outcomes (Figure 8(c)).

## 4. Discussion

The present research performed bioinformatics analysis of two profile datasets (GSE17025 and GSE63678) to identify more useful prognostic biomarkers in EC. This study included 17 normal endometrial tissues and 98 EC tissues. A total of 249 common DEGs (adjusted *P* value < 0.05 and ∣log_2_FC | >1), of which 102 were downregulated (log_2_FC < ‐1) and 147 were upregulated (log_2_FC > 1), were found through GEO2R and the Draw Venn Diagram. Next, DAVID was applied to analyse GO and KEGG pathway enrichment. The results showed that (1) among the BP terms, upregulated DEGs were mainly enriched in cell division, mitotic nuclear division, G2/M transition of mitotic cell cycle, sister chromatid cohesion, mitotic sister chromatid segregation, and anaphase-promoting complex-dependent catabolic process and downregulated DEGs were enriched in the regulation of transcription from RNA polymerase II promoter, transcription, DNA-templated, response to glucocorticoid, positive regulation of cardiac muscle cell proliferation, negative regulation of transcription from RNA polymerase II promoter, and mast cell migration; (2) among the CC terms, upregulated DEGs were markedly enriched in cytosol, midbody, nucleus, spindle pole, condensed chromosome kinetochore, and spindle and downregulated DEGs were enriched in the nucleus and cytoplasm; (3) among the MF terms, upregulated DEGs were enriched in protein binding, ATP binding, microtubule binding, microtubule motor activity, protein kinase binding, ATP-dependent microtubule motor activity, and plus-end-directed and downregulated DEGs were enriched in transcription factor activity, sequence-specific DNA binding, nucleic acid binding, RNA polymerase II transcription factor activity, sequence-specific DNA binding, metal ion binding, sequence-specific DNA binding, and chromatin binding. In the KEGG pathway analysis, upregulated DEGs were significantly enriched in cell cycle, p53 signalling pathway, biosynthesis of antibiotics, oocyte meiosis, carbon metabolism, and glycolysis/gluconeogenesis, while downregulated DEGs were enriched in pathways in cancer, signalling pathways regulating pluripotency of stem cells, melanoma, acute myeloid leukaemia, transcriptional misregulation in cancer, and proteoglycans in cancer (*P* < 0.05). Then, we constructed a DEG PPI network complex of 2551 edges and 197 nodes by using STRING and the Cytoscape app. Next, through Cytotype MCODE analysis, 64 crucial upregulated DEGs were selected from the PPI network complex. Furthermore, 14 of 64 genes exhibited associations with significantly worse survival via UALCAN analysis. GEPIA analysis, which was used to validate these 14 genes, showed that all of these genes were more highly expressed in EC samples compared with normal endometrial samples (*P* < 0.01). DAVID was applied to reanalyse the KEGG pathway enrichment of these 14 genes. The enrichment of three genes (*TTK*, *CDC25A*, and *ESPL1*) in the cell cycle pathway was significant (*P* < 0.05). Lastly, *TTK*, *CDC25A*, and *ESPL1* showed higher expression in cancers with late stage and higher tumour grade, which indicates that they may be potential targets for improving EC patient prognosis.

*TTK*, also known as monopolar spindle1 (*Mps1*), was discovered during a screening of spindle pole body replication genes in 1991 and is a kinetochore-localized protein kinase with dual specificity [22, 23]. *TTK* is essential for mitotic checkpoints and participates in cell survival and the repair of oxidative DNA lesions. High *TTK* expression may help tumours overcome the challenges of an oxidative microenvironment [24, 25]. Many studies have shown that *TTK* is overexpressed in several human malignant tumours, including malignant melanoma (MM), non-small cell lung carcinoma (NSCLC), prostate cancer, breast cancer, and colon cancer. Its expression is associated with the poor patient outcome. Knockdown of *TTK* or treatment with a *TTK* inhibitor could suppress tumour growth by inhibiting cell proliferation, migration, and tumorigenesis. Thus, *TTK* may be a potential therapeutic target for cancers [26–30].

Cell division cycle 25 (*CDC25*) is a bispecific phosphatase that removes phosphate groups from phosphorylated serine (Ser, S), threonine (Thr, T), and tyrosine (Tyr, Y) residues of its substrate proteins [31]. As a member of the *CDC25* phosphatase family, *CDC25A* is essential for the progression of the cell cycle from G1 to S phase. *CDC25A* is involved in a variety of biological processes, such as G1/S transition, cell division, regulation of cyclin-dependent protein serine/threonine kinase activity, cell proliferation, regulation of cell cycle, DNA replication, and cellular response to UV [32, 33]. Considered an oncogene, *CDC25A* is highly expressed in many types of human malignancies, such as breast cancer, ovarian cancer, head and neck cancer, colon cancer, and cutaneous squamous cell carcinoma [34–38]. Moreover, *CDC25A* expression was significantly associated with tumour invasion and poor tumour differentiation [39].

*ESPL1* (extra spindle pole-like 1) is an endopeptidase and cysteine protease. *ESPL1* is activated and cleaves the cohesin subunit *RAD21* to release a sister chromatid cohesion required for chromosomal disjunction at the onset of anaphase [40–42]. *ESPL1* is introduced to mitotic chromosomes to dissolve the cohesion of the sister chromatid in a DNA-dependent manner, playing an important part in the progression of the cell cycle, ensuring faithful genetic inheritance [43, 44]. Several studies have shown that *ESPL1*, as a candidate oncogene, is overexpressed in several types of breast cancers [45–47]. It is also a marker for mitotic activity and prognosis in breast cancers [48].

As discussed above, several studies have suggested that these three genes are associated with the progression of many types of cancer. However, our search of the PubMed database reveals that very few studies have investigated the roles of these three genes in EC. Thus, this study can provide helpful information and direction for future studies of EC.

## 5. Conclusions

In summary, we identified three DEGs (*TTK*, *CDC25A*, and *ESPL1*) between normal endometrial tissues and EC tissues in our bioinformatics analysis based on two mRNA expression datasets. These three genes might play important roles in the development of EC. A series of future experiments will be needed to validate these predictions. Nevertheless, these data can provide helpful information and direction for the elucidation of potential biomarkers and the biological mechanisms of EC.

## Figures and Tables

**Table 1:** All 249 DEGs were detected from two mRNA expression datasets, of which there are 147 upregulated genes and 102 downregulated genes in the EC.

DEGs	Gene names
Upregulated	ESPL1, E2F8, DHCR24, VAMP8, TSTA3, ANXA1, TPX2, CCNB1, GOT1, ANP32E, LMNB2, KPNA2, PDIA3, BIRC5, TAGLN2, FOXM1, F2RL1, CDK1, CHEK1, CARMIL1, MUC1, KIF11, GPI, MRPS12, PDXK, EZR, CDC6, HSPA4, CENPU, PCCB, SMC4, APOBEC3B, AURKA, CLDN7, KIF14, ANXA2P2, MAD2L1, ATP1A1, TJP3, FANCA, BLM, KIF4A, KRT8, LDHA, SCD, MAP7, MPZL1, UCK2, KIF2C, EIF5A, ANP32A, ACLY, TYMS, MELK, SFN, VDAC1, CDC20, CENPN, HN1, ZWINT, MPDU1, SAR1A, CCNA2, GTSE1, PBK, TRIP13, S100A11, STAT1, PTTG1, MMP12, CDC7, CKS2, ISG15, ECT2, KIF23, ANXA2, GSR, TK1, CENPE, ASPM, UBE2S, LMNB1, SPAG5, CDCA3, CKMT1B, PTBP3, TACC3, UBE2C, CCNB2, PRC1, LRP8, CKAP2, CEP55, PLEKHB2, RRM2, MGST2, CENPA, TOP2A, SYNCRIP, FEN1, RBM47, MOB1A, KIF18A, KIF15, ST14, SSR1, BUB1B, S100A10, DLGAP5, HJURP, RAD51AP1, ESRP1, ENO1, MKI67, DTL, GDE1, SULF1, NCALD, ACP1, RAD51, HMMR, TUBB4B, CDCP1, ARF3, KIF20A, MIF, GMDS, SDF2L1, IDE, CTSZ, DLAT, GAPDH, KIAA0101, MTCH2, TTK, PGD, CDKN3, NME1, NCAPG, MYCBP, BAX, CIT, NEK2, CENPF, NUSAP1, PGK1, CDC25A
Downregulated	H3F3B, HYMAI, NAALAD2, GNAL, MITF, HOXD11, ERG, EVC, HNMT, CA11, GHR, ROR2, KLF3-AS1, BCHE, SRSF11, WT1-AS, POU6F1, LEFTY2, PHF1, RBPMS, ZNF667, GABBR1, VSTM4, SPAG9, RUNX1T1, PER1, KIAA0368, PPIEL, TRPC4, SNCA, TSPYL5, ENPP2, UNKL, SOX15, CTSF, NR2F2, ZDHHC17, PNISR, TCEAL2, FOXN3, KIAA1644, PKD1P1, FBXO9, WNT2, MCOLN3, STAT5B, ENPEP, CBX7, ARMCX1, TBX3, FAM184A, ZFP2, CACNB2, PEG3, HAND2-AS1, C2orf68, TGFBR3, ZNF37BP, MAF, ZNF135, PGM5, ATRNL1, ST3GAL5, BNC2, AKT3, CYP1B1, EFS, CDIP1, ZSCAN18, KLF11, ZNF506, MXRA8, UBE2I, TRO, C1orf21, PLAGL1, BHMT2, ST8SIA1, GATAD1, PAK3, PDS5B, NMT2, CMAHP, MAGEH1, H3F3A, EZH1, CDO1, NUDT11, GSPT2, HNRNPDL, FGF2, CXCL12, IGF1R, CRBN, GPRASP1, MAGEL2, CHRD, ME3, CIRBP, NUMA1, SNED1, TNS2

## Data Availability

The data used to support the findings of this study are included within the article.
